# Correspondence: Space-time asymmetry undermines water yield assessment

**DOI:** 10.1038/ncomms11603

**Published:** 2016-05-12

**Authors:** Wouter R. Berghuijs, Ross A. Woods

**Affiliations:** 1 Department of Civil Engineering, University of Bristol, University Walk, Bristol BS8 1TR, UK

Understanding the effects of climate and land-cover changes on water yield is a challenging component in assessments of future water resources. Zhou *et al*.^1^ use Fuh's equation^2^, based on the widely used Budyko framework^3^, to quantify the spatial differences of mean water yield normalized by precipitation (*R/P*) as a function of the climate wetness index (precipitation/potential evaporation *P/PET*) and watershed characteristics (*m*). Similar to what has been done before^4^, Zhou *et al*.^1^ subsequently derive the sensitivity of *R/P* to both wetness (∂*R/P*/∂*P/PET*) and watershed characteristics (∂*R/P*/∂*m*) to expose the role of climatic and land-use changes on water yield. However, Zhou *et al*.^1^ ignore several crucial assumptions that undermine their use of the framework.

First, Zhou *et al*.^1^ analyse how three variables (*R/P, P/PET, m*) co-vary in space, to approximate their behaviour in time, and thereby implicitly assume that there is symmetry between spatial (between-watershed) and temporal (between-years) partitioning of precipitation into streamflow and evaporation. This is not necessarily the case for watersheds^5^. To test if this symmetry assumption is valid we compare ∂*R/P/*∂*P/PET*, as approximated by the Zhou *et al*.^1^ equation (see Methods equation 1, which is based on spatial differences), and the ∂*R/P/*∂*P/PET* calculated using inter-annual water balances (see Methods equation 2, which is based on temporal differences). Figure 1 shows a scatterplot of the spatial vs. temporal sensitivities for the widely used MOPEX data set^6^,^9^ consisting of 420 watersheds located in diverse climates and landscapes. The sensitivity metrics are significantly correlated (*R*^2^=0.502, *P*-value<0.001), but on average the difference between the two metrics is 27.9%. There are distinct regional patterns showing to what degree the spatial and temporal sensitivities differ (Fig. 1). This suggests that in certain landscapes space is tradable for time. However, in other landscapes, this assumption leads to systematic under- or overestimation of the water yield's sensitivity to climate change. Why there are regional differences between spatial and temporal precipitation partitioning, and how these differences can change when the landscape coevolves with climate are open questions that still need to be answered^7^.

Second, Zhou *et al*.^1^ attribute any effects of *m* to landscape characteristics. However, both empirical evidence and modelling studies^6^,^8^,^9^ indicate that climate intra-annual variability is a major factor in determining the *R/P* of a watershed, and thereby it also strongly affects *m*. When Roderick and Farquhar^4^ first introduced the sensitivity framework, they acknowledged that this watershed parameter encodes all factors other than climate wetness that change the partitioning of precipitation between evaporation and streamflow. Therefore *m* also includes the effects of precipitation seasonality, timing, intensity and form (for example, snow vs. rain). Ignoring this role of climate intra-annual variability can bias the attribution of *m* towards landscape properties, prevents landscape effects from being strictly separated from intra-annual climate effects, overestimates the importance of landscape effects, and ignores the role of part of the climate effects on water yield.

Third, Zhou *et al*.^1^ claim to identify the critical values of *P/PET* and *m* that define ranges where either climate or landscape changes are more important for water yield. In addition to the above-identified issues of the framework, the so-called ‘critical values' given in Zhou *et al*.^1^ are misleading. Sensitivity to wetness (∂*R/P/*∂*P/PET*) and watershed characteristics (∂*R/P/*∂*m*) are both dimensionless metrics and therefore potentially comparable. However, comparison of these metrics is not meaningful for assessing water yield changes (Δ*R/P)* unless they are combined with typical changes of climate (Δ*P/PET*) and watershed parameter (Δ*m*). Additionally, the wetness index can vary by orders of magnitude between watersheds (see Fig. 7 in ref. 1). Because climate change (Δ*P/PET*) is proportionally related to the occurring wetness index, a 10% increase in precipitation can lead to an order of magnitude difference in Δ*P/PET* values. The methods and conclusions on the relative importance of climate and landscape sensitivity given in Zhou *et al*.^1^ do not take these aspects into account. The authors are therefore not able to identify the real relative importance of climate and landscape on water yield.

Finally, Zhou *et al*.^1^ state that the pattern of *m*, *P/PET* values and their correlation with landscape properties can explain the diverse effects of forest cover changes on water yield. This implies that Fuh's equation can predict both negative and positive streamflow changes in response to increase in forest cover; however, this implication is not correct. The proposed explanation for positive sensitivity is independent of Fuh's equation. Zhou *et al*.^1^ instead rely on their assertion that *PET* over forests is lower than *PET* over grassland in the same location, because of the lower temperatures observed over forests^10^. However, the use of any *PET* equation that relies only on temperature is clearly inappropriate for this question, since forests and grasslands have very different partitioning of energy between latent and sensible heat. Lower temperatures over forests are not sufficient to estimate changes in forest *PET*, and even if they were, this would not be a consequence of Fuh's equation; as a result we do not agree that Zhou *et al*.^1^ have provided an explanation for cases where water yield from forest exceeds that from grassland.

In summary, we disagree with the main conclusion of Zhou *et al*.^1^ that their study exposes the relative roles of climate and landscape in water yield. Four major issues, all related to the asymmetry of temporal and spatial conditions, constrain the current validity of the framework. First, data indicate that the central assumption of tradability of space and time is not valid for all landscapes. Second, the landscape parameter *m* is, using the authors' approach, not separable from important and ignored intra-annual climate conditions. Third, the critical values of *P/PET* and *m* identified by Zhou *et al*.^1^ only provide mathematical guidance on the importance of climate and landscape, but the connection with real-world changes is still to be clarified. Finally, the framework does not provide the claimed explanation for increases in water yield associated with increases in forest cover. We therefore recommend acknowledging these limitations and emphasize that a unifying framework of the water yield's sensitivity to climate and landscape changes needs to be more conservative in its assumptions, or needs to better address space-time asymmetry of the co-variation of *P/PET* and *R/P*, climate intra-annual variability, and typical rates of change of climate and landscape.

## Methods

### Water yield sensitivity calculations

Zhou *et al*.^1^ used the partial derivative of Fuh's equation to calculate the water yield sensitivity to wetness index:


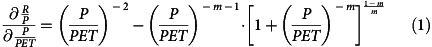


where *P*, *PET* and *m* denote the long-term average values of precipitation, potential evaporation and watershed characteristics. We calculate water yield sensitivity to climate based on temporal differences using the slope (*α*):


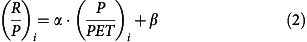


where 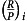 is the water yield of year *i*, and 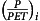 is the wetness index of year *i*. The *α* value is approximated by the slope terms of least-squares estimators. Annual values used in the analysis are from 1 September to 31 August to minimize the effects of carry-over of water storage. Repeating the analysis for 5-year values yields similar results.

## Additional information

**How to cite this article:** Berghuijs, W. R. & Woods, R. A. Correspondence: Space-time asymmetry undermines water yield assessment. *Nat. Commun.* 7:11603 doi: 10.1038/ncomms11603 (2016).

## Figures and Tables

**Figure 1:**
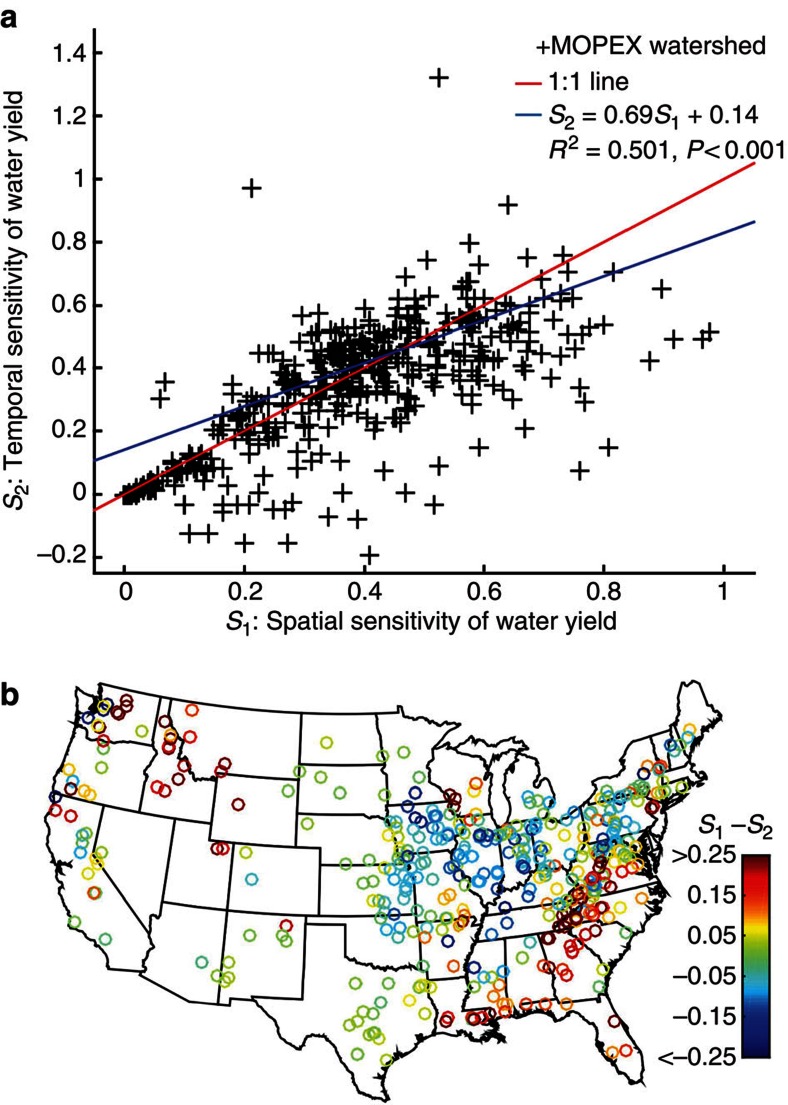
Spatial and temporal sensitivity of water yield. Spatial sensitivity of water yield (x-axis) and temporal sensitivity of water yield (y-axis) for 420 watersheds located in the United States (**a**), and the spatial pattern of their differences (**b**).
